# Diet as a Modulator of Intestinal Microbiota in Rheumatoid Arthritis

**DOI:** 10.3390/nu12113504

**Published:** 2020-11-14

**Authors:** Eduardo Dourado, Margarida Ferro, Catarina Sousa Guerreiro, João Eurico Fonseca

**Affiliations:** 1 Serviço de Reumatologia e Doenças Ósseas Metabólicas, Centro Hospitalar Universitário Lisboa Norte, Centro Académico de Medicina de Lisboa (CAML), 1649-028 Lisboa, Portugal; 25546@chln.min-saude.pt; 2 Unidade de Investigação em Reumatologia, Instituto de Medicina Molecular, Faculdade de Medicina, Universidade de Lisboa, CAML, 1649-028 Lisboa, Portugal; 3 Laboratório de Nutrição, Faculdade de Medicina, Universidade de Lisboa, 1649-028 Lisboa, Portugal; margarida.ferro97@gmail.com (M.F.); cfguerreiro@medicina.ulisboa.pt (C.S.G.); 4 Instituto de Saúde Ambiental, Faculdade de Medicina, Universidade de Lisboa, 1649-028 Lisboa, Portugal

**Keywords:** rheumatoid arthritis, gut microbiota, Mediterranean diet, probiotics

## Abstract

Rheumatoid arthritis (RA) is a chronic immune-driven inflammatory disease characterised by synovial inflammation, leading to progressive cartilage and bone destruction, impacting patients’ functional capacity and quality of life. Patients with RA have significant differences in gut microbiota composition when compared to controls. Intestinal dysbiosis influences the intestinal barrier strength, integrity and function, and diet is considered the main environmental factor impacting gut microbiota. Over the last few years, researchers have focused on the influence of single components of the diet in the modulation of intestinal microbiota in RA rather than whole dietary patterns. In this review, we focus on how the Mediterranean diet (MD), a whole dietary pattern, could possibly act as an adjuvant therapeutic approach, modulating intestinal microbiota and intestinal barrier function in order to improve RA-related outcomes. We also review the potential effects of particular components of the MD, such as n-3 polyunsaturated fatty acids (PUFAs), polyphenols and fibre.

## 1. Introduction

Rheumatoid arthritis (RA) is a chronic immune-driven inflammatory disease characterised by joint swelling, joint tenderness, destruction of synovial joints and systemic inflammation, ultimately causing severe disability and premature mortality [1,2,3,4]. Early mortality has been largely attributed to an increased rate of cardiovascular (CV) events that is independent of traditional CV risk factors [5,6,7] and associated with increased systemic inflammation [6,7]. Patients with a persistently high disease activity have a higher rate of CV events [8] and higher mortality risk [3,7].

Over the last two decades, the optimal use of disease-modifying anti-rheumatic drugs (DMARDs) and the advent of biologic therapies as well as novel small molecules have dramatically enhanced the success of RA management, improving the quality of life and decreasing the mortality of RA patients [1,3,8,9,10], primarily due to reduced CV mortality [9].

However, a substantial proportion of patients with RA still cannot achieve remission with pharmacological therapies alone, irrespective of the remission definition used [11]. As a consequence, several non-pharmacological adjuvant therapies are currently being explored, including patient education, exercise therapy and other physical modalities, orthoses, assistive devices, balneotherapy and dietary interventions [12].

The global health improvement potential of dietary interventions in RA patients cannot be understated. Even if one should disregard the potential benefit of diet in RA inflammatory activity, dietary interventions could still have a significant impact on both the CV risk and the prevalence of obesity, a known risk factor for a poor quality of life [13,14,15], comorbidities [13] and treatment resistance [14,15,16] in RA patients.

It has been proposed that the Mediterranean diet (MD), together with genetic and other lifestyle factors, could explain the lower incidence of RA in Southern Europe compared to Northern Europe and North America [17,18]. This, however, is still controversial, since studies have failed to prove that adherence to the MD is associated with a decreased risk of developing RA [19,20].

The MD constitutes a set of skills, knowledge, practices and traditions, including crops, harvesting, fishing, conservation, processing, preparation and, in particular, the consumption of food [21]. It is characterised by a nutritional model consisting mostly of olive oil, cereals, fruit and vegetables, a moderate amount of fish, dairy and meat, and many condiments and spices, accompanied by wine, sugar-free herbal infusions and tea, and low-sodium and low-fat broths [21,22].

Several mechanisms have been proposed to explain the influence of the MD in RA prevalence and disease activity, including increased antioxidant levels, lipid profile alteration with a shift towards an anti-inflammatory profile, and gut microbiota modulation [23].

More recently, developments in the field of pharmacomicrobiomics have revealed the role of gut microbiota on the pharmacokinetics of (and individual response to) immunomodulatory drugs [24,25], unveiling yet another potential benefit of gut microbiota modulation in RA patients.

In this paper, we review the potential role of the MD as adjuvant therapy in RA management, with a focus on diet–gut microbiota interactions.

## 2. Gut Microbiota and Rheumatoid Arthritis

### 2.1. Gut Microbiota Composition

The human gut houses the largest population of non-eukaryotic organisms in the human body. Despite the high variability between individuals’ gut microbiota composition, bacteria are consistently (and by far) the most common microorganisms [26,27,28]. *Bacteroidetes* and *Firmicutes* are the most abundant phyla [26,27,29]. Significant inter-individual differences in the prevalence of certain species within these phylae have been reported, resulting from different genetic backgrounds, dietary habits, lifestyle, hygiene practice, drug use and other environmental factors [29,30,31]. The composition of the gut microbiome in people living in distinct geographical areas has a strong association with each population’s dietary habits [27,31].

Urbanisation has been closely related to a Western dietary style, antibiotic use and pollution. It induces changes in gut microbiota composition, particularly the loss of intra-individual microbial diversity accompanied by higher inter-individual differences [32]. In non-Westernised communities, people tend to have a more homogeneous and diverse gut microbiome [32,33,34]. Their gut microbiota tends to be rich in certain bacteria, such as *Bacteroidetes* (including *Prevotella* and *Xylanibacter*), and poor in *Firmicutes* [31,32,34].

Three different classes of enterotypes have been proposed according to the abundance of *Bacteroides*, *Prevotella* and *Ruminococcus*, respectively [27]. There is a strong association between the individual enterotype and long-term but not short-term diet [35].

A healthy microbiota is characterised by the presence of numerous classes of bacteria, with a balanced composition of symbiont and pathobiont organisms [36]. A pathobiont is a permanent resident of the microbiota that does not usually elicit an inflammatory response, but under particular environmentally-induced conditions has the potential to cause dysregulated inflammation and induce disease [36]. A shift in the microbiota composition, with either an increase in pathobionts or a reduction in symbionts, leads to a state of dysbiosis [36] that disturbs the modulation of the host immune function by the gut microbiota [30,36].

### 2.2. Gut Microbiota, Mucosal Immune System and Intestinal Permeability

The mucosal immune system and intestinal microbiota can influence each other, promoting a balance between tolerance to dietary antigens and protection against harmful pathogens [29,30,37]. Antigen-presenting cells located at the mucosal surface, once activated by antigens, can regulate immune tolerance by promoting T cell differentiation into regulatory T cells (Tregs) [38]. Tregs suppress inappropriate activation of effector T cells by secreting anti-inflammatory cytokines [29,39]. The gut microbiota influences the number and function of colonic Tregs [40], suggesting that the modulation of gut microbiota may also regulate the mechanism of gut immune tolerance. Most importantly, these interactions may also modulate systemic inflammation, and influence the risk of developing systemic autoimmune diseases [41], including inflammatory arthropathies [41].

The production of short-chain fatty acids (SCFAs) is one of the proposed mechanisms by which intestinal microbiota affects Treg cells differentiation and systemic inflammation [42]. SCFAs, particularly butyrate, acetate and propionate, are the key metabolites resulting from the microbial fermentation of dietary fibres [32,42,43,44]. When fermentable fibres are in short supply, bacteria switch to energetically less favourable sources for growth, such as amino acids or dietary fats [45], resulting in the reduced fermentative activity of the microbiota and SCFA scarcity [42]. SCFAs are an energy source for gut epithelial cells, having an indirect anti-inflammatory effect by improving the assembly of tight junctions and enhancing intestinal barrier function [32,46]. Butyrate is the preferred energy source for colonocytes and is locally consumed, whereas other absorbed SCFAs drain into the systemic circulation [42]. Histone acetylation is thought to increase accessibility to the transcriptional machinery, to promote gene transcription. Butyrate and propionate are known to act as histone deacetylase inhibitors [47]. Through this mechanism, SCFAs may act as systemic anti-inflammatory or immune-suppressive molecules [42]. Despite the low concentration in the periphery, propionate and butyrate affect peripheral organs indirectly by activation of the hormonal and nervous systems [42].

The modulation of intestinal permeability is another mechanism that may explain the influence of gut microbiota on the appearance and perpetuation of inflammatory diseases [48], leading to systemic inflammation. The intestinal lumen is occupied by various exogenous constituents such as microorganisms, toxins and food antigens. The mucosal barrier, which separates the intestinal milieu from the luminal environment, has an essential role in blocking the entry of microorganisms and toxins, while, at the same time, it must allow the absorption of nutrients and water [49,50]. Intestinal barrier strength and function can be affected by several factors, among which diet, gut microbiota composition and mucosal immune system integrity are key factors [51,52,53,54]. When the mucosal barrier is disturbed, the exogenous luminal constituents invade the intestinal milieu, and immune activation and mucosal inflammation ensue [39,48,55]. This process can trigger an abnormal immune response resulting in both local and systemic inflammation, increasing the risk of developing both gut-associated and extra-intestinal diseases [48,51,54,56].

### 2.3. Gut Microbiota and Rheumatoid Arthritis Pathogenesis

Over the last few years, emerging evidence has reported the involvement of gut dysbiosis in the onset of autoimmune diseases such as RA [29,30,57], suggesting its role in contributing to a breakdown of immune tolerance [49,51,58,59].

The natural history of RA includes different stages, including an at-risk pre-symptomatic phase and an early arthritis phase before the classical erosive disease phase [29,58,60,61,62]. The most noticeable players in the preclinical RA phases are the autoantibodies, a hallmark of RA [29,60]. RA-associated autoantibodies include rheumatoid factor (RF) [63,64], antibodies to citrullinated proteins (ACPA) [65] and anti-carbamylated peptide antibodies (anti-CarP) [66]. High serum concentrations of RF [67], ACPA [68], and anti-CarP [69] can be detected years before the onset of clinically overt RA. The evolution of the autoantibody profile in the preclinical stage of RA includes isotype switching [29]. The higher prevalence of IgA- than IgG-ACPA in high-RA-risk populations suggests that mucosal immune responses are important in the preclinical phases of the disease [29]. In different groups of individuals who later developed RA, a higher prevalence of serum IgA- and IgM-RF than IgG-RF was found, and IgA-RF appeared earliest [70].

Growing evidence suggests that the dysbiosis of mucosal microbiota is closely related to local autoimmune processes [71], and the composition of the microbiota is significantly disturbed in patients with both early and long-standing RA [72]. It is also known that dysbiosis in the oral microbiota induces periodontitis [73] and that the gingiva of patients with periodontitis contains citrullinated proteins and ACPA [74,75]. *Porphyromonas gingivalis*, a common periodontal pathogen, can citrullinate targets for ACPA [76], suggesting that dysbiosis and periodontitis can play a fundamental role in the early loss of tolerance to self-antigens in RA-susceptible patients [59,77,78].

Different mechanisms can explain the dysbiosis-mediated induction of autoantibodies. The most common theory states that T helper cells can originate from T cell responses to external antigens through molecular mimicry between those antigens and self-antigens [29,79,80]. Such activation leads to the positive selection and maturation of self-reactive B cells that produce a variety of RA-associated autoantibodies [79,81,82]. It has also been proposed that host cell necrosis and apoptosis, occurring as a consequence of bacterial insult, can result in extracellular exposure of intracellular self-antigens, leading to recognition by B cells and autoantibody production [82].

Recently, it has been shown that the gut microbiota of RA patients has a significant increase in the class of *Bacilli* and the order of *Lactobacillales* compared to healthy controls [57,83]. This is concordant with data reporting an increase in the *Lactobacillaceae* family and the *Lactobacillus* genus in mice susceptible to collagen-induced arthritis [84]. The variety of *Lactobacilli* is also higher in RA patients [83]. Excess *Lactobacillus salivarius* in the gut and mouth of RA patients has been described, and a correlation with disease severity has been proposed [85]. Paradoxically, the administration of *Lactobacillus acidophilus* and *Lactobacillus casei* (*L. casei*) seems to be beneficial for RA disease activity (further discussed in Section 4.1), suggesting that different *Lactobacilli* probably have different roles in RA pathogenesis and disease activity modulation [86,87].

On the other hand, significant reductions of the genus *Flavobacterium*, *Faecalibacterium* and other butyrate-producing taxa [57,88,89], as well as its related species *Faecalibacterium prausnitzii* and the species *Blautia coccoides* were described [55].

Moreover, a higher prevalence of Euryarchaeota, Gammaproteobacteria, Pasteurellales, and Anaerobranca zavarzinii was correlated with a higher disease activity score-28 (DAS28), whilst Erysipelotrichi, Erysipelotrichales, Coriobacteriales, Coriobacteriaceae, Lactobacillaceae, Collinsella, Bacteroides rodentium, and Collinsella aerofaciens were inversely associated with this score [57].

Importantly, it has also been shown that RA patients under treatment with etanercept, a fusion protein consisting of a human tumour necrosis factor (TNF) receptor linked to the Fc portion of human immunoglobulin G1 (IgG1), present a partial restoration of a beneficial microbiota [57]. This fact seems to provide further evidence pointing towards gut dysbiosis as a hallmark of the disease.

### 2.4. Gut Microbiota and Drug Metabolism

Pharmacomicrobiomics is an emerging field that investigates the effect of variations within the human gut microbiome on drugs [24]. The variability between individuals in the composition and metabolic competence of their microbiomes has a unique role in determining the bioavailability, clinical efficacy and toxicity of a wide array of drugs, including DMARDs [24]. This variability arises because specific, direct modifications of the chemical structures of ingested drugs are dependent on the composition of the human gut microbiome and its collective enzymatic activity [90,91].

Relevant examples include the prodrug sulfasalazine, which requires cleaving by the gut microbiome to become an active drug [92], as well as cyclophosphamide and methotrexate [24]. Although our basic understanding of how microbiome-dependent biotransformations of xenobiotics affect human health is still incomplete, numerous studies have highlighted the extent to which microbial xenobiotic metabolism varies between individuals, and how these reactions can be manipulated for therapeutic purposes [24,93,94]. Diet modifications and probiotics are the biggest candidates to play this modulatory role.

## 3. Mediterranean Diet as a Modulator of Gut Microbiota in Rheumatoid Arthritis

Environmental factors, rather than genetic ones, are predominant in changing the composition of gut microbiota [31,32]. Diet is one such environmental factor and is considered a major determinant of gut microbiota composition [32,95]. The modulation of gut microbiota through nutritional factors is increasingly recognised as a potential interventional approach for the prevention and management of several diseases, including RA [32,96].

As previously stated, the MD has a food consumption pattern characterised by the extensive use of olive oil, cereals, fruit and vegetables, a moderate amount of fish, dairy and meat, and many condiments and spices [21] that replace the use of salt [97,98].

The MD is frequently recommended to patients with chronic inflammatory diseases [97] since it has anti-inflammatory and antioxidant properties [98,99,100] with the potential to modulate inflammatory pathways [98,100]. Interestingly, n-3 polyunsaturated fatty acids (PUFAs), monounsaturated fatty acids (MUFAs), dietary fibre and polyphenols are recognised as key components of the MD [23].

### 3.1. Mediterranean Diet, Polyunsaturated Fatty Acids and Rheumatoid Arthritis

Linoleic acid (LA) and alfa-linolenic acid (ALA) are essential fatty acids, meaning the human body cannot synthesise them. LA and ALA are n-6 and n-3 PUFA precursors, respectively. LA and n-6 PUFAs most typically originate from animal sources, whilst ALA is mainly produced by green-leafed vegetables [101], and n-3 PUFAs can be acquired from oily fish, poultry, nuts and berries [102].

LA and ALA are essential components of phospholipid membranes, and both are precursors of inflammatory mediators, such as prostaglandins (PGs) and leukotrienes (LTs) when metabolised to eicosanoids. The n-6 PUFAs and LA have a pro-inflammatory profile, whilst n-3 PUFAs and ALA have a role in the resolution of the inflammatory response [18,98]. The same enzymes metabolise both LA and ALA, with greater affinity for the anti-inflammatory ALA [103].

Because its most typical foods contain n-3 PUFAs, the MD has a balanced n-6/n-3 PUFA ratio, unlike the Western diet, which is characterised by a deficiency in n-3 and excess n-6 PUFA consumption [98,104]. This high n-6/n-3 PUFA ratio shifts the enzymatic activity towards n-6 PUFA usage [105] and is associated with a highly pro-inflammatory profile.

Studies suggest that n-3 PUFAs have a protective role in RA-susceptible patients [106], although several issues remain unclear regarding their actual role in RA pathogenesis [107]. This relationship has been extensively investigated, and the anti-inflammatory and pro-resolving effects of the n-3 PUFAs seem to justify this association [107].

Dietary interventions targeting PUFAs have been designed [98]. Fish oil, containing mainly eicosapentaenoic acid (EPA) and docosahexaenoic acid (DHA), is the primary source of n-3 PUFAS used in clinical interventions [98]. However, MD may be a more reasonable intervention targeting the n-6/n-3 PUFA ratio.

### 3.2. Mediterranean Diet, Gut Microbiota and Short-Chain Fatty Acids

**Figure 1:**
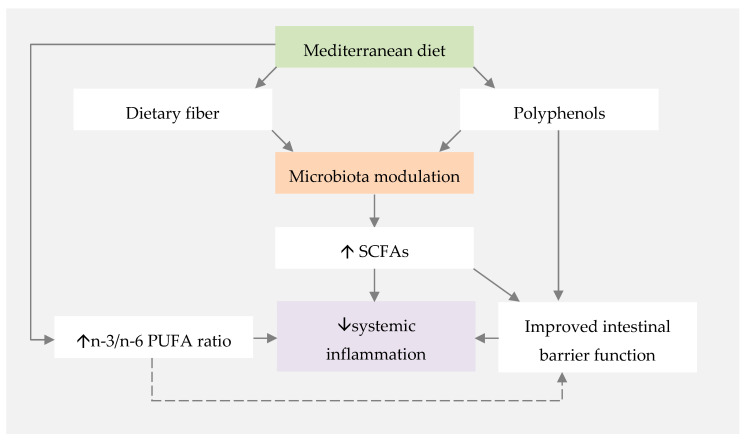
Proposed mechanisms for the influence of the Mediterranean diet on systemic inflammation. PUFA—polyunsaturated fatty acids; SCFAs—short-chain fatty acids.

The influence of the MD on gut microbiota composition and its effect on SCFA production has been widely studied, given the interest in the anti-inflammatory properties of SCFAs (Figure 1). Different studies reported that higher adherence to the MD is associated with a more diverse microbiota and higher levels of faecal SCFA [108,109,110]. This diversity may be associated with the MD’s richness in plant-based foods, and, therefore, dietary fibre [43].

Carbohydrates’ effects on intestinal microbiota depend not only on the type of carbohydrates ingested but also their relative amounts. Generally, a diet richer in carbohydrates leads to a gut abundance of Bacteroidetes, including Prevotella species, producers of SCFA [111]. Dietary fibres are defined as nondigestible carbohydrates that are available to the colonic microbial community [43]. Interestingly, plant-based foods provide several different types of dietary fibre. Some fibres have fermentable properties, making them an important energy source for microbial populations. These fibres stimulate the growth of some microbes, having a prebiotic-like function [43,112], and modulating the gut microbiota composition [95].

Gutierréz-Díaz et al. investigated the association between regular adherence to the MD and the faecal microbiota composition among adults with established dietary habits. Higher adherence to the MD was associated with higher concentrations of Bacteroidetes and Firmicutes, as well as higher concentrations of faecal propionate and butyrate [113]. Garcia-Mantrana et al. reported that individuals with a higher MD score had a higher proportion of Bacteroidetes and a lower Firmicutes/Bacteroidetes ratio [109], suggesting that Bacteroidetes are the most favoured phyla. In a different study by Mitsou et al., higher adherence to the MD was associated with lower levels of faecal *E. coli*, higher Bifidobacteria/*E. coli* ratio, and higher total SCFA [108]. These results support the idea that regular adherence to the MD modulates gut microbiota and increases SCFA production.

A systematic review focusing on the effect of dietary fibre interventions on gut microbiota composition in healthy adults described an increase in *Bifidobacterium* and *Lactobacillus* spp., as well as higher faecal butyrate concentration with dietary fibre interventions [95].

Another mechanism that may justify the increased production of SCFAs in individuals that are adherent to the MD is the high intake of polyphenols. Polyphenols are widely present in plant-based foods and beverages, including fruits, vegetables and red wine [114,115], all of which are abundantly present in MD. It is increasingly recognised that health benefits credited to polyphenols are related to gut microbiota composition modulation [115]. The effects on gut microbiota include the promotion of symbionts’ survival and multiplication, as well as the inhibition of pathogenic bacteria replication, profoundly modulating the gut micro-ecology [115,116]. Results from a study investigating the association between the MD and microbial derived-phenolic compounds in faecal samples showed that individuals with higher adherence to the MD have a significant increase in microbiota levels of Clostridium cluster XIV and *Faecalibacterium prausnitzii*, both recognised as essential producers of butyrate in the human colon [117,118].

Olive oil, which is rich in polyphenols and MUFAs, represents the primary source of fat of the MD [99,119]. Extra virgin olive oil (EVOO) has potent antioxidant, anti-inflammatory and immunomodulatory effects, mainly due to its content of phenolic compounds [100,120].

### 3.3. Diet and Gut Permeability Modulation

Studies on how to modulate gut permeability are now arising. The impact of different dietary components on gut permeability has still been poorly studied. Dietary strategies that theoretically support the gut barrier function include: (i) the avoidance of energy-dense Western diets, sugars and fat; (ii) the use of prebiotic- and probiotic-rich diets; and (iii) diets that increase the SCFA production [53], such as the MD. In animal studies, butyrate strengthens the barrier through the increase in TJ components ZO-1, ZO-2, and cingulin [121].

Moreover, there is increasing evidence suggesting that polyphenols improve the intestinal barrier function [52,120,122,123]. Polyphenol-rich diets have beneficial effects on intestinal barrier function both by direct mechanisms, such as higher expression of TJ proteins like ZO-1 and clausin-1 and by indirect mechanisms, such as gut microbiota modulation [124].

The role of n-3 PUFAs in the modulation of gut permeability is debated, with reports stating they can improve intestinal barrier resistance and reduce interleukin (IL)-4-mediated permeability [125], and reports stating that the intestinal permeability is increased [126,127].

Recent findings by Di Palo et al. showed that subjects with lower adherence to the MD tend to have increased intestinal permeability [128], confirming the notion derived from the previously mentioned studies.

Bearing all these findings in mind, new strategies to improve intestinal barrier strength may include (i) fibre-rich diets with high intakes of plant-based foods, (ii) polyphenol sources, such as olive oil, and (iii) adequate intake of n-3 PUFAs, commonly achieved through a higher intake of oily fish, to improve the n-3/n-6 PUFA ratio [129]. Interestingly, these are some of the key components of the MD, suggesting that the adoption of the MD may be a feasible approach to modulate and improve the intestinal barrier function.

### 3.4. Mediterranean Diet and Rheumatoid Arthritis Disease Activity

The beneficial effects of the MD in health and disease have been widely reported, but only a small number of trials have focused on RA [98]. Although research in the field of diet–health relationships is changing its paradigm, and the study of whole dietary patterns is becoming more pertinent [130], more relevance has been given to the impact of specific components in RA-related outcomes [98].

Several studies evaluated the effects of n-3 PUFAS on clinical outcomes of RA [131,132,133]. Higher EPA and DHA intakes have been shown to increase the presence of these components in cell membranes, replacing arachidonic acid (AA) in the phospholipid bilayer of immune cells. This reduces the cells’ pro-inflammatory propensity, and EPA and DHA act as substrates for the synthesis of pro-resolving mediators, with an essential role in the mechanisms of recovery of homeostasis after inflammation [134,135]. Abdulrazaq et al. conducted a systematic review investigating the role of n-3 PUFAS on arthritic pain. Among the 18 included randomised controlled trials (RCTs), 10 sustained the hypothesis that a decrease in pain is obtained after an intervention with n-3 PUFAs [136]. Several studies have also reported a positive effect of n-3 PUFAs on other clinical outcomes of RA, namely a reduction in the duration of morning stiffness (MS), and a decrease in tender (TJ) and swollen joints (SJ) counts [137]. A meta-analysis conducted by Gioxari et al., confirmed that oral intake of n-3 PUFAs resulted in significant improvements of MS, TJ count, erythrocyte sedimentation rate (ESR) and visual pain scale [133]. Although only a small number of studies evaluated the Health Assessment Questionnaire (HAQ), grip strength and Richie Articular Index (RAI), these parameters also improved significantly [133]. Meta-analyses by Gaioxari et al. and Jiang et al. reported a significant decrease in leukotriene B4 (LTB4) levels with n-3 PUFAs supplementation in RA patients [133,138]. Jiang et al. did a subgroup analysis and found a significant decrease in LTB4 levels in patients with RA but not in patients with chronic non-auto-immune diseases [138]. LTB4 is an inflammatory lipid mediator with a relevant role in the activation and recruitment of leukocytes to the inflamed areas [139]. The excessive formation of LTB4 leads to an exacerbated inflammatory response, inducing chronic inflammation in diseases such as RA. High serum and synovial fluid levels of this eicosanoid can be found in patients with active RA [133,139]. Lee et al. conducted another meta-analysis and reported a consistent result of the n-3 PUFAS effect in reducing EMS, SJ count and visual pain scale [140]. The need for non-steroidal anti-inflammatory drugs (NSAIDs) also decreased when compared to placebo [140]. Despite all of the data supporting the positive effects of n-3 PUFAs in RA, heterogeneity regarding the n-3 PUFAS dosage and intervention duration, as well as the use of different components as the placebo, represent limitations to these studies. A dosage of PUFAs (EPA and DHA) of at least 3 g/day over at least 12 weeks seems to be required to obtain significant improvements in the visual pain scale in RA [136].

It has been suggested that EVOO may have a synergetic effect with other components of the MD in RA-related outcomes. Patients with RA taking a daily intake of fish oil for 24 weeks, a significant source of n-3 PUFAs, seemed to have greater improvements in disease-related symptoms when combined with EVOO than with fish oil alone [141].

Data from the “(The TOtal Management Of Risk factors in Rheumatoid arthritis patients to lOWer morbidity and mortality” (TOMORROW) study revealed an inverse correlation between the consumption of MUFA/SCFA and DAS28-ESR [142]. Moreover, a higher intake of MUFAs acted as an independent factor influencing disease remission [142].

Two RCTs evaluated the direct effect of MD in RA-related outcomes. Sköldstam et al. conducted a RCT to evaluate the effect of a Cretan MD versus a standard Western diet on RA disease activity. Results showed that patients with RA with stable and low disease activity obtained significant improvements in DAS-28, HAQ, and two components of short-form-36 after 12 weeks of intervention with a Cretan MD [143]. However, several flaws of this study can be pointed out. The sample size was small, and some patients had DAS-28 as high as seven, i.e., high disease activity, at the beginning of the intervention. Furthermore, patient body mass index (BMI) was statistically different between the two groups at the start of the intervention, and the analysis was only performed at 12 weeks after the intervention started, without more extended follow-up data. In a different interventional study, Mckellar et al., compared written information and cooking classes as means of MD implementation in women with RA. Intake of fruit, vegetables and legumes increased significantly over three months, and the use of monounsaturated compared with saturated fats also improved in the workshops group [144]. A significant benefit was shown in the intervention group compared with controls for PGA at six months, pain score at three and six months, MS at six months and HAQ at three months [144]. These results suggest that the more effective the learning method of the MD, the greater can the achieved results be. Although this study’s results were statistically significant, the improvements were modest, and DAS-28 did not significantly improve [144]. Therefore, confirmation of these data is required, preferably with larger sample sizes.

## 4. Beyond the Mediterranean Diet: The Role of Probiotics and Fermented Foods

Probiotics are living microorganisms that can improve an individual’s health when administered in adequate amounts [145]. Prebiotics are a group of nutrients that are degraded by the gut microbiota, supporting the survival of symbionts [146]. The administration of probiotics and prebiotics unsurprisingly impacts gut microbiota composition and functionality [147].

Probiotics have immunomodulatory effects not only via direct immune system modulation, but also through the regulation of intestinal permeability and competition with pathobionts [147]. Multiple studies with different strains, such as *Lactobacillus rhamnosus* and *Lactobacillus casei* (*L. casei*), proved that probiotics modulate the expression and distribution of tight junctions’ proteins ZO-1 and occludin [147]. *Lactobacillus* and *Bifidobacterium* genus are recognised as producers of SCFA, resulting in the enforcement of the gut barrier function [148].

### 4.1. Probiotics in Rheumatoid Arthritis

Zamani et al. investigated the effect of an eight-week probiotic supplementation in both the disease activity and the metabolic status of RA patients [149]. Participants in the probiotics group used capsules containing a mixture of *Lactobacillus acidophilus, L. casei* and *Bifidobacterium bifidum*. This group of patients had improvements in DAS28, high-sensitivity C-reactive protein (hs-CRP) serum levels and lower levels of serum insulin versus placebo [149].

An RCT conducted by Vaghef-Mehrabany et al. evaluated the effects of daily supplementation with *L. casei* for eight weeks in disease activity and inflammatory cytokines levels in RA patients [87]. *L. casei* supplementation resulted in an improved DAS28 and a significant reduction in CRP at the end of the trial. Additionally, there was a reduction in the levels of pro-inflammatory cytokines such as TNF, IL-6 and IL-12, and an increase in the levels of IL-10, a known anti-inflammatory cytokine [150].

Alipour et al. evaluated the effects of *L. casei* supplementation for eight weeks on disease activity and serum inflammatory biomarkers in female patients with RA [86]. Significant improvements were observed in TJ- and SJ-counts, hs-CRP, and DAS28 in the probiotic group [86]. A decrease in the levels of the inflammatory cytokines was also observed [86].

In 2018, a meta-analysis evaluated the net effect of probiotics supplementation on RA patients and included the four available RCTs versus placebo published [151]. In this study, the effectiveness of probiotic supplementation in improving disease-related outcomes in RA patients was considered weak [151]. A recent review by Oliviero et al. has highlighted that the effect of probiotic supplementation in RA seems to be specie- and strain-specific [152].

### 4.2. Fermented Foods, Probiotics and Short-Chain Fatty Acids

In the past few years, the interest in fermented foods has boomed in Western countries, due to its proposed health benefits [153]. Fermented foods include both foods and beverages that are produced under controlled microbial growth, making use of the conversion of food components through microbial enzymatic action [154]. Fermented foods include *Kefir*, *Kombucha*, *Sauerkraut, Tempeh*, *Natto*, *Miso*, *Kimchi* and *Sourdough bread* [154]. *Bifidobacterium* and *Lactobacillus* genera can be found in several of these fermented foods [153].

**Figure 2:**
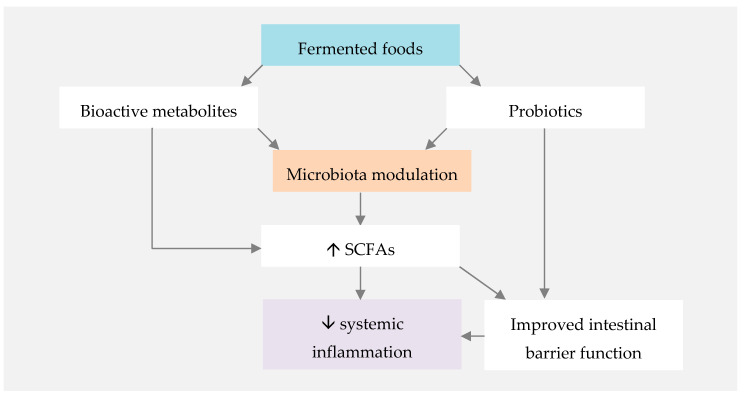
Proposed mechanisms for the influence of fermented foods on systemic inflammation. SCFAs—short-chain fatty acids.

Observational and interventional human studies suggest that fermented foods can have a protective role against metabolic and immune-mediated disorders [155]. The mechanism by which fermented foods promote human health is related to both their probiotic content and their bioactive metabolites, formed in the fermentation process (Figure 2) [156]. SCFA, whose benefits have been previously highlighted in this paper, are among such bioactive metabolites [155].

Although the evidence in this field is still limited, the massive potential of including fermented foods in a healthy whole-dietary pattern such as the MD ensures that further investigation will ensue. Furthermore, we have to take into account that the similar anti-inflammatory pathways of both these dietary components may potentiate each other, making their combination appealing for RA patients.

## 5. Conclusions

The MD’s health benefits are well documented. However, evidence regarding its impact on RA disease activity is still insufficient, and diet is still not considered in RA management recommendations.

**Figure 3:**
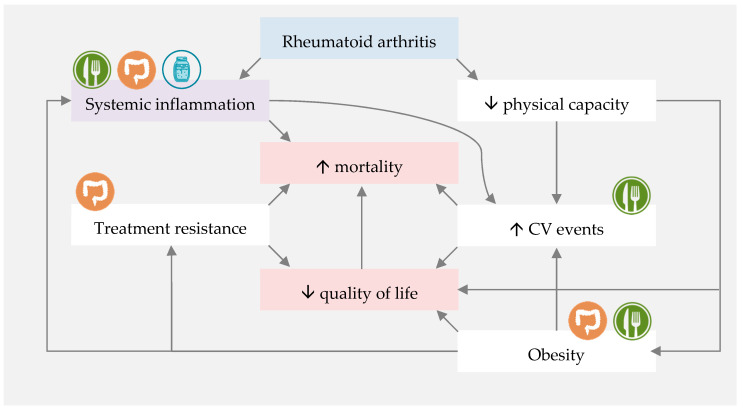
Proposed mechanisms of Mediterranean diet (MD) and fermented foods influences on rheumatoid arthritis-associated quality of life and mortality. 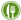 represents direct influence of MD or nutrients. 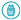 represents direct influence of fermented foods. 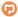 represents indirect MD and fermented foods influence through gut microbiota modulation. CV—cardiovascular.

However, based on current knowledge, introducing a diet which is rich in n-3 PUFAs, polyphenols, dietary fibre and probiotics may decrease systemic inflammation, modulate gut microbiota and improve the gut barrier function, and consequently improve RA disease outcomes. The MD fits these assumptions, and fermented foods seem to complement it perfectly, adding probiotics and active metabolites to an almost ideal dietary pattern for RA patients (Figure 3).

Moreover, pharmacomicrobiomic investigation is currently unveiling the role of microbiota on DMARD treatment responses, making gut microbiota modulation in RA a clear hot topic.

These new data warrant further exploration of the MD, complemented or not with fermented foods, as a potential adjuvant therapy for RA patients, ideally in well-designed studies with large sample sizes and a multidisciplinary team of researchers, including rheumatologists and nutritionists.
